# Untargeted metabolomics profiling of gestational diabetes mellitus: insights into early diagnosis and metabolic pathway alterations

**DOI:** 10.3389/fmolb.2024.1485587

**Published:** 2024-12-23

**Authors:** Shereen M. Aleidi, Hiba Al Fahmawi, Reem H. AlMalki, Maha Al Mogren, Mohammad Alwahsh, Muhammad Mujammami, Michele Costanzo, Anas Abdel Rahman

**Affiliations:** ^1^ Department of Biopharmaceutics and Clinical Pharmacy, School of Pharmacy, The University of Jordan, Amman, Jordan; ^2^ Department of Medicinal Chemistry, College of Pharmacy, University of Sharjah, Sharjah, United Arab Emirates; ^3^ Metabolomics Section, Department of Clinical Genomics, Center for Genomics Medicine, King Faisal Specialist Hospital and Research Centre (KFSHRC), Riyadh, Saudi Arabia; ^4^ Department of Pharmacy, Faculty of Pharmacy, Al-Zaytoonah University of Jordan, Amman, Jordan; ^5^ Endocrinology and Diabetes Unit, Department of Medicine, King Saud University, Riyadh, Saudi Arabia; ^6^ University Diabetes Center, King Saud University Medical City, King Saud University, Riyadh, Saudi Arabia; ^7^ Department of Molecular Medicine and Medical Biotechnology, School of Medicine, University of Naples Federico II, Naples, Italy; ^8^ CEINGE-Advanced Biotechnologies Franco Salvatore scarl, Naples, Italy; ^9^ Department of Biochemistry and Molecular Medicine, College of Medicine, Alfaisal University, Riyadh, Saudi Arabia

**Keywords:** gestational diabetes, pregnancy, untargeted metabolomics profiling, biomarkers, diagnosis

## Abstract

**Introduction:**

Gestational Diabetes Mellitus (GDM) is a metabolic disorder marked by Q10 hyperglycemia that can negatively affect both mothers and newborns. The increasing prevalence of GDM and the limitations associated with the standard diagnostic test highlight the urgent need for early screening strategies that promote timely interventions.

**Methods:**

This study aims to investigate the metabolic profile associated with GDM through an untargeted metabolomic analysis using mass spectrometry (MS)- based omics. Serum samples were collected from 40 pregnant women at weeks 24–28 of gestation based on the 2-h 75-g oral glucose tolerance test (OGTT); 50% were diagnosed with GDM (n = 20), and the remaining were considered a control group.

**Results and discussion:**

The results showed distinct metabolic differences between women with GDM and those without, with 222 significantly dysregulated metabolites, 120 up- and 102 downregulated in GDM compared to the control group. Key metabolic pathways, such as tryptophan, inositol phosphate, phenylalanine, and histidine metabolism, were notably dysregulated in GDM. The study also found that specific metabolites, like N-Acetylproline and Serylmethionine, with area under the curve (AUC) of 0.978 and 0.968, respectively, showed high accuracy in distinguishing between GDM and non-GDM women. This study would enhance our understanding of metabolic alterations in GDM and could contribute to early prediction and management strategies.

## 1 Introduction

GDM arises from hormonal changes during pregnancy when the placenta releases hormones that decrease the cell’s responsiveness to insulin (Choudhury and Devi Rajeswari, 2021). It is characterized by glucose intolerance, first recognized during pregnancy (Choudhury and Devi Rajeswari, 2021; Wicklow and Retnakaran, 2023). It poses significant risks to the mother and the developing fetus, including neonatal hypoglycemia, macrosomia, and an increased likelihood of metabolic complications later in life (Wicklow and Retnakaran, 2023).

The diagnosis of GDM is based on the 24–28-week OGTT (Lachmann et al., 2020). However, this test has limitations, including expense, inconvenience (Lachmann et al., 2020), and limited sensitivity in early pregnancy (Huhn et al., 2023). GDM affects approximately 14% of pregnancies worldwide, with varying prevalence rates depending on the population (Wang H. et al., 2022). The increasing prevalence of GDM and the limitations associated with the standard OGTT highlight the urgent need for early screening strategies that promote timely interventions that could potentially improve maternal and neonatal outcomes (Bhattacharya et al., 2021; Razo-Azamar et al., 2023). Such strategies include potential predictive biomarkers that are sensitive, specific, and capable of detecting GDM early in pregnancy (Razo-Azamar et al., 2023).

Metabolomics, an emerging analytical approach, offers a sensitive and comprehensive method for identifying changes in the levels of metabolites with a molecular weight of less than 1,500 Da in a specific biological sample (Siddiqui et al., 2020). Our recent review highlighted the crucial role of metabolomics in advancing the understanding of the molecular mechanisms and metabolic pathways implicated in Type 1 and Type 2 diabetes (Aleidi et al., 2023). Metabolomics also holds promising potential as a screening tool for GDM, though evidence on this issue is limited and needs adequate assessment through randomized controlled trials (Razo-Azamar et al., 2023). By analyzing the metabolic profile of biological samples, such as plasma or urine, metabolomics offers a holistic view of metabolic alterations associated with GDM pathogenesis (Francis et al., 2023).

Previous metabolomic studies have identified candidate biomarkers and revealed predictive models for the early diagnosis of GDM. However, many of these studies are limited by employing targeted metabolomic approaches, focusing on specific metabolites rather than providing a comprehensive view of the metabolic profile in GDM (Razo-Azamar et al., 2023; Juchnicka et al., 2022; Mokkala et al., 2020). Furthermore, there is insufficient validation of biomarkers across pregnant women with different body mass indexes (BMI) (Mokkala et al., 2020). These limitations highlight the need for more comprehensive studies to uncover the metabolic alternations in GDM and improve clinical outcomes for affected pregnant women. This study aims to investigate the metabolic alterations associated with GDM in pregnant women through an untargeted metabolomic analysis using MS technology of serum samples collected from GDM patients and matched controls. The results of this study would provide a detailed view of the serum metabolome in GDM. In addition, they would contribute to the growing body of evidence implicating specific metabolic pathways in GDM pathophysiology and identify novel biomarkers for early diagnosis and intervention.

## 2 Methods

### 2.1 Study population and selection criteria

This cross-sectional study was conducted in the obstetrics and gynecology outpatient clinic at a hospital in Jordan. Forty pregnant women aged 18–40 years at weeks 24–28 of gestation were included in this study. Twenty of them were diagnosed with GDM (patient group) based on the 2-h 75-g (OGTT, and the other twenty were non-GDM and considered a control group. Diagnosis of GDM was considered according to the American Diabetes Association (ADA) criteria, in which pregnant women who have any of the following criteria (fasting blood glucose (FBG) ≥92 mg/dL, 1-h ≥180 mg/dL or 2-h ≥153 mg/dL) were diagnosed with GDM. All pregnant women included in this study did not have pre-eclampsia and proteinuria, and this was confirmed by considering patients’ history on blood pressure readings and urinalysis retrospectively. Women who have prediabetes, type 1 or 2 diabetes mellites, dyslipidemia, polycystic ovary syndrome (PCOS), and renal impairment were excluded from the study.

### 2.2 Sample collection, storage, and preparation

Blood samples were collected from all participants and centrifuged at 1,500×g for 10 min at 4°C. The obtained serum was transferred into micro tubes and stored at − 08°C until the day of analysis. Metabolite extraction was conducted from serum following an extraction protocol previously published (Dahabiyeh et al., 2023). Cold methanol and chloroform were added to 35 μL serum followed by water and shaking. Equal volumes of chloroform and water were added before centrifugation at 10,000×g for 5 min. All samples were dried using a vacuum centrifugal evaporator and stored at − 80°C until further analysis.

### 2.3 Metabolomic analysis

All dried extracted samples were reconstituted in 50% mobile phase A (0.1% formic acid in deionized water) and mobile phase B (0.1% formic acid in (1:1) (v/v) methanol and acetonitrile) for an LC-MS metabolomics analysis as previously reported (AlMalki et al., 2023). Initially, 5 μL of the reconstituted sample was introduced to the inlet technique, where the metabolites were separated in a reversed-phase liquid chromatography with Waters ACQUITY UPLC XSelect C18 (100 × 2.1 mm × 2.5 μm) column (Waters Ltd., Elstree, United Kingdom). The mobile phase flow rate was set to 300 μL/min, and the column was maintained at 55°C while the sample was stored at 4°C in the autosampler. Mobile phases A and B were pumped in a gradient mode as follows: 95%–5% A (0–16 min), 5% A (16–19 min), 5%–95% A (19–20 min), and 5%–95% A (20–22 min). The eluted molecules from the column were ionized in the electrospray ionization source (ESI) at positive and negative modes. The gas phase ions were subjected to Xevo G2-S QTOF mass spectrometer (Waters Ltd., Elstree, United Kingdom) separation based on their m/z. The MS source temperature was fixed at 150°C, the desolvation temperature was set at 500°C, and the capillary voltages were kept at 3.20 kV or 3 kV for ESI+ and ESI− modes, respectively. The cone gas flow was 50 L/h, the desolvation gas flow was 800 L/h, and the cone voltage was 40 V. The collision energies for the low and high functions were set to off and 10–50 V, respectively, in the MS^E^ data-independent acquisition (DIA) mode. As recommended by the vendor, the mass spectrometer was calibrated with sodium formate (100–1,200 Da) in both ionization modes. The lock spray mass compound, MS leucine-enkephaline (an external reference to the ion m/z 556.2771 in positive mode and 554.2615 in negative mode), was constantly injected, which is responsible for switching between the sample and the reference for every 45 and 60 s in both modes, scan time was 0.5 s, the flow rate was 10 μL/min, and collision energy was 4 V and 30 V for the cone, respectively. The DIA data were gathered in continuum mode with Masslynx™ V4.1 Software (Waters Inc., Mil-ford, MA, United States). Quality control samples (QCs) were performed by collecting 10 µL from each study sample and pooling them for extraction. After that, they were introduced to the instrument randomly to validate the system’s stability (AlMalki et al., 2023). After that, they were analyzed following the routine protocol. The acceptance criteria were to have all the QC samples separated from the other study groups, clustered together, and use their Relative standard deviations (RSD%) < 40%

### 2.4 Data and statistical analysis

The MS raw data were processed following a standard pipeline starting from alignment based on the m/z value and the ion signals’ retention time, peak picking, and signal filtering based on the peak quality using the Progenesis QI v.3.0 software from Waters (Waters Technologies, Milford, MA., United States). Multivariate statistical analysis was performed using MetaboAnalyst version 5.0 (McGill University, Montreal, Canada) (http://www.metaboanalyst.ca, accessed on 10 March 2024) (Pang et al., 2021). The data sets (Compounds’ names and their raw abundances) were median-normalized, Pareto-scaled, and log-transformed to maintain their normal distribution. The normalized datasets generated partial least squares-discriminant analysis (PLS-DA) and orthogonal partial least squares-discriminant analysis (OPLS-DA) models. OPLS-DA models were evaluated using the fitness of model (R2Y) and predictive ability (Q2) values using permutation validation of 100 samples (Worley and Powers, 2013). Univariate analysis was performed using Mass Profiler Professional (MPP) v.15.0 software (Agilent, Santa Clara, CA, United States). A volcano plot was used to discover significantly changed mass features based on a Moderated T-test, cut-off: FDR *p* < 0.05, fold change 2 compared to controls (Gu et al., 2020). Heatmap analysis for altered features was performed using Pearson distance measure according to the Pearson similarity test. Pathway analysis, biomarkers linked with GDM, and receiver operating characteristic (ROC) curves were generated in the MetaboAnalyst (v.5.0) by Monte Carlo cross-validation (MCCV) with balanced sub-sampling. During each MCCV iteration, two-thirds (2/3) of the samples were designated for assessing feature importance. The top-ranked features, as determined by the Partial Least Squares Discriminant Analysis (PLS-DA) algorithm, were subsequently employed to construct classification models. These models were then validated on the remaining one-third (1/3) of the samples. This procedure was iteratively performed multiple times to ascertain each model’s performance and confidence interval. The PLS-DA algorithm was utilized for feature ranking with latent variables (LV) 2. The value was only considered if the provided LV count was within the number of features. Clinical characteristics and demographic data were presented either as categorical data (frequency) or continuous data (mean ± standard deviation (SD)). Data were analyzed using SPSS Software version 24. To compare the between the two groups of the study, chai square or independent sample *t*-test were used, considering significant P values less than 0.05.

### 2.5 Metabolites identification

All the statistically significant features between the study groups were selected using Progenesis QI v.3.0 software (Waters Technologies, Milford, MA, United States) for peak annotation (Aleidi et al., 2021; Dahabiyeh et al., 2021). The precursor and product ions were annotated based on accurate mass, fragmentation pattern, and isotopic distributions in the Human Metabolome Database (HMDB) with a 5-ppm mass error (Wishart et al., 2022) and 5 ppm for METLIN MS/MS (https://metlin.scripps.edu/) accessed 2 April 2024, using fragmentations filtered by *in silico* or empirical, KEGG, Lipid map, and Lipid Blast. Exogenous metabolites, such as food additives, pharmaceuticals, and exposome molecules, were removed from the final list.

## 3 Results

### 3.1 The clinical characteristics and demographic data of the study population

Table 1 presents the clinical characteristics and demographics of the study population. The control and GDM groups were matched in age, BMI, gestational weeks, and complete blood count (CBC) tests. Based on the study design and group definitions, there was a significant difference between control and GDM in the FBG and both 1 h PG and 2 h PG.

**TABLE 1:** The clinical characteristics and demographics of the study population (n = 40).

Parameter	Control (n = 20)	GDM (n = 20)	P-value
Age (years)	32.3 ± 5.51	32 ± 6.38	0.87
Gestational age (week)	25.89 ± 2.62	25.25 ± 2.57	0.43
Gestational weight gain (kg)	7.85 ± 4.23	8.3 ± 5.96	0.79
BMI (based on current weight) (kg/m2)	31.56 ± 7.44	31.03 ± 5.13	0.80
FBG (mg/dL)	79.40 ± 6.20	92.53 ± 7.44	<0.001
1 h-PG (mg/dL)	122.15 ± 29.93	179.05 ± 40.55	<0.001
2 h-PG (mg/dL)	93.55 ± 20.79	131.35 ± 31.67	<0.001
Hemoglobin (Hb) (g/dL)	11.62 ± 1.49	11.58 ± 1.15	0.97
Hematocrit (Hct) (%)	34.37 ± 4.01	33.90 ± 6.52	0.78
Red blood cells (RBC) (10^3^/µL)	5.71 ± 6.79	7.35 ± 9.47	0.52
White blood cells (WBC) (10^3^/µL)	10.25 ± 4.25	10.69 ± 6.42	0.80
Platelet (10^3^/µL)	261.42 ± 62.62	246.84 ± 79.89	0.51
Fetal gender Male/Female undetermined	(8/9) 3	(15/3) 2	0.03
Mode of previous deliveries Vaginal/C-sections	(11/9)	(15/5)	0.14

### 3.2 The comprehensive metabolomic analysis and contrasts among the study groups

**FIGURE 1:**
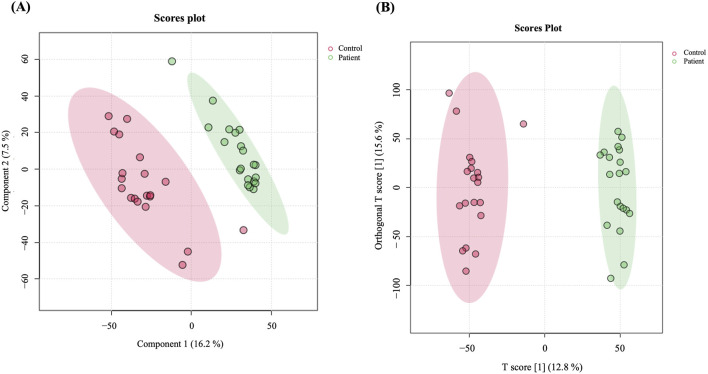
Metabolomics profiling of GDM patients compared to controls. **(A)** PLS-DA displaying separation between groups. **(B)** OPLS-DA model shows evident separation between GDM and Control. The robustness of the created models was evaluated by the fitness of the model (R2Y = 0.976) and predictive ability (Q2 = 0.822) values in a larger dataset (n = 100).

A total of 19,736 mass ion features were detected after reviewing the signal quality with intensity >1,000 counts, 13,374 in positive and 6,362 in negative ionization modes (Supplementary Table S1). The features used for analysis were 11,568 after excluding the missing values with a frequency of 80% of each study groups, which were statistically evaluated between the study groups (GDM and controls). The metabolomics profiles of study groups were analyzed using the PLS-DA model (Figure 1A). This model was used to explore the clustering and differentiation among the groups. Figure 1A illustrates a separation in the metabolic profiles between the control and GDM patients.

Furthermore, an OPLS-DA model was utilized to compare GDM patients and control groups, visualizing the plot’s classification effect, clustering, and separation. The model demonstrated a clear, evident, and significant separation between the two groups (R2 = 0.976 and Q2 = 0.822, Figure 1B), indicating variations in metabolite expression between women with GDM and the control group.

### 3.3 Metabolomics profiling of GDM and control groups

**FIGURE 2:**
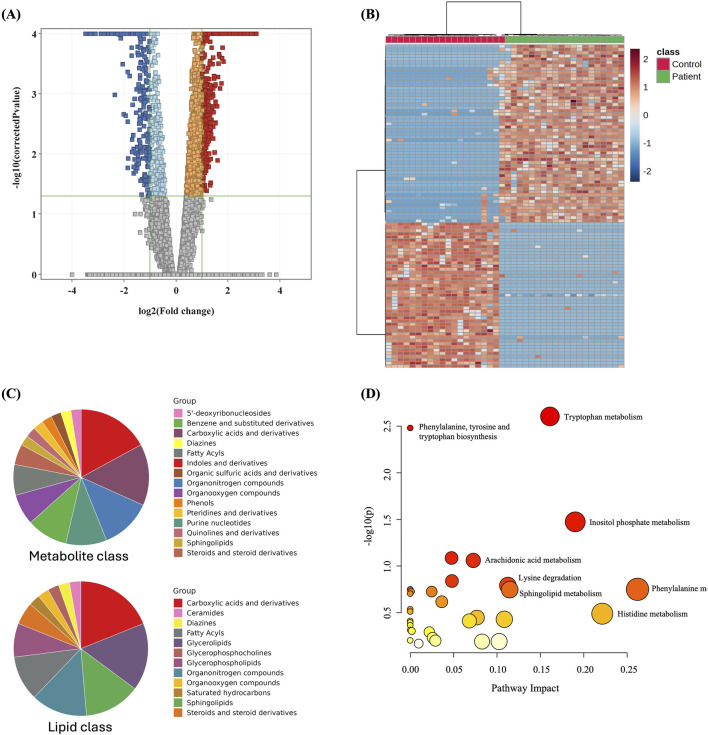
Dysregulated metabolites between GDM patients and control and pathway analysis **(A)** Volcano plot shows significantly dysregulated metabolites in GDM Patients compared to Control; shows 1,003 metabolites were significantly dysregulated, where 607 and 396 metabolites were up (red) and down (blue)-regulated in GDM compared to Control, respectively. **(B)** Heat map showing the distribution of the dysregulated metabolites between GDM and control groups. **(C)** Main metabolites and lipids classes of the significantly dysregulated human endogenous metabolites (n = 222). **(D)** Pathway analysis of significantly dysregulated endogenous metabolites (n = 222) in GDM patients compared to controls.

The metabolomics profiling of GDM and control groups was statistically evaluated using volcano plot analysis considering *t*-test moderated, FDR *p* < 0.05, and Fold Change (FC) cut-off 2. The results revealed that 1,003 metabolites were significantly dysregulated between the study groups, Figure 2A. Among them, 607 and 396 were up- and downregulated in GDM patients compared to the control group (Figure 2A, Supplementary Table S2). Among the significantly dysregulated (n = 1,003), 553 were annotated using HMDB, METLIN MS/MS, LipiBlast, LipidMap, and KEGG (Supplementary Table S3). After excluding exogenous metabolites, such as drugs, drug metabolites, or environmental exposure-related metabolites, 222 were identified as human endogenous metabolites of which 120 were up- and 102 were downregulated between the two groups (Supplementary Table S4, human endogenous metabolites). The main metabolites and lipids classes of the dysregulated endogenous metabolites (n = 222) are presented in Figure 2C. Moreover, metabolic pathway analysis revealed that the most relevant metabolic pathways related to the dysregulated metabolites included tryptophan metabolism, inositol phosphate metabolism, phenylalanine metabolism, and histidine metabolism (Figure 2D).

### 3.4 Biomarker evaluation

**FIGURE 3:**
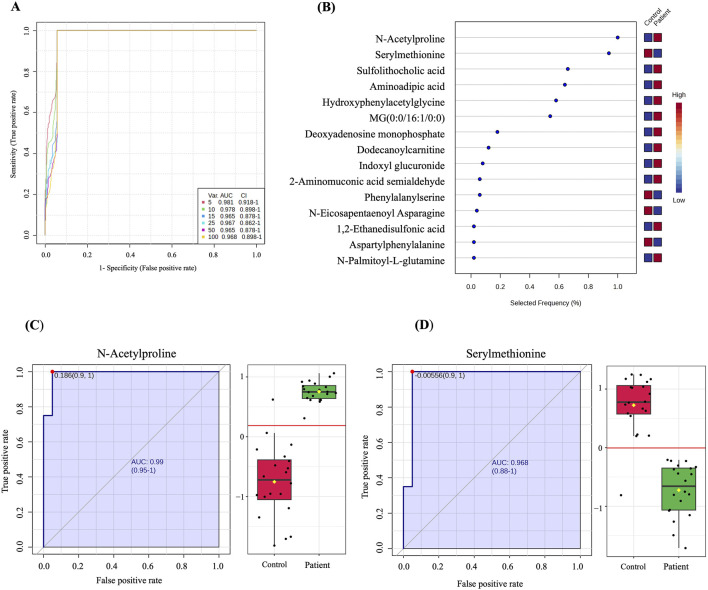
Biomarker evaluation between GDM patients and controls. **(A)** A ROC curve was generated by the OPLS-DA model, with AUC values calculated from the combination of 5,10, 15, 25,50, and 100 metabolites. **(B)** The frequency plot shows 15 positively identified significant dysregulated endogenous metabolites between GDM patients and control. **(C, D)** Representative AUC for two significantly dysregulated endogenous metabolites. **(C)** N-Acetylproline, AUC = 0.99, upregulated) and **(D)** Serylmethionine, AUC = 0.968, downregulated in GDM patients compared to controls.

A multivariate exploratory ROC analysis based on the identified significantly dysregulated human endogenous metabolites between GDM patients and controls (n = 222) was generated using OPLS-DA as a classification and feature ranking method. Combining the top 10 metabolites in the exploratory ROC curves indicates the maximum confidence of differentiation and detection of metabolites in the GDM versus control group, with the AUC = 0.978, Figure 3A. The significant features of the positively identified metabolites are presented in Figure 3B. Furthermore, representative AUCs for two dysregulated metabolites in GDM compared to controls are shown in Figures 3C, D. These include N-Acetylproline, AUC = 0.99, upregulated in GDM, and Serylmethionine, AUC = 0.968, downregulated in GDM compared to controls.

## 4 Discussion

GDM, a prevalent metabolic disorder during pregnancy, is characterized by glucose intolerance in the second and third trimesters, leading to hyperglycemia (Wang et al., 2021). Indeed, early diagnosis of GDM and managing blood glucose levels are instrumental in significantly alleviating patient complications. OGTT remains the gold-standard diagnostic tool for GDM diagnosis. However, the recognition of more sensitive and accurate approaches for earlier detection of this disorder is important. Recent methods based on machine-learning algorithms seem to discretely predict GDM development in the first trimester even though the accuracy of the trained models may not be elevated (Wang, Huang, 2024), thus increasing the risk of false positives or negatives. Consequently, finding new early biomarkers is imperative, and metabolomics approaches retain their full potential for such goals (Santorelli et al., 2023).

Metabolomics research in GDM focuses on abnormalities in small molecule metabolites such as carbohydrates, lipids, amino acids, bile acids, sterol hormones, and altered metabolic pathways (Wang et al., 2021). The current study applied an untargeted MS-based metabolomics approach to investigate the pregnancy metabolic fingerprint related to GDM. The detected metabolic signature is mainly characterized by alterations in amino acid metabolism, fatty acid, glycerolipids, and sphingolipids that distinguish GDM from control women. These altered metabolites could be used as potential biomarkers aiding in disease diagnosis, monitoring, and discovering new therapeutic targets, providing new insights into the pathophysiology of GDM.

By multivariate statistical analysis, including PLS-DA and OPLS-DA, our results show a clear group separation and sample clustering between GDM and control groups. Tryptophan metabolism, inositol phosphate metabolism, phenylalanine metabolism, and histidine metabolism are among the pathways that have been significantly altered between GDM and controls. It is well known that the high inflammatory state in GDM alters amino acid metabolism, including tryptophan metabolism (Chatree et al., 2020). Inositol phosphate metabolism is directly connected to insulin resistance and glucose homeostasis (Chatree et al., 2020). Pregnant women with GDM exhibit much greater insulin resistance than those with normal glucose tolerance. Ellerbrock et al. found that one in every two women with GDM has severe insulin resistance, compared to one in every five with normal glucose management (Ellerbrock et al., 2022). The disturbances in amino acid metabolism are closely linked to insulin resistance, as demonstrated by the histidine and phenylalanine metabolism pathways observed in this study.

Several omics-based studies have revealed that circulating metabolites belonging to diverse chemical classes (e.g., lipids, fatty acids, amino acids, acylcarnitines, *etc.*) are positively associated with the incidence of GDM in either early or mid-pregnancy (Wang et al., 2021; Alesi et al., 2021; Lin et al., 2021; Zhao et al., 2019; Zhu et al., 2018), with either common identifications or contrasting findings. Our results show that with various differentially expressed metabolites, most of the metabolites attributed to GDM were dipeptides, amino acids, and their derivatives with branched amino acids (BCAAs), such as the decrease of L-isoleucine, L-histidine, 5-hydroxy-DL-tryptophan, and methionine-conjugated molecules in GDM, being in line with other published metabolomics studies (Burzynska-Pedziwiatr et al., 2020; Enquobahrie et al., 2015; Meng et al., 2021). Previous studies have found a significant association between levels of several amino acids and GDM (Wang et al., 2021; Liu et al., 2021; Lu et al., 2021). Some studies indicate that high plasma BCAA levels relate to an increased risk of GDM (Lu et al., 2021). Indeed, BCAA metabolism is involved in pathways that sustain the energetic metabolism (Costanzo et al., 2024) and modulate insulin resistance, thus affecting insulin secretion (Newgard, 2012). Our present study showed a significant decrease in L-isoleucine levels in GDM; a possible explanation might be increased consumption of amino acids such as isoleucine by the placenta and fetus in GDM (Wang X. et al., 2022).

Our findings indicate that histidine is downregulated in GDM. Histidine is an essential amino acid involved in protein synthesis and the function of enzymes (Ingle, 2011). Spanou et al. reported that histidine levels were significantly reduced in women with GDM compared to controls (Spanou et al., 2022). This reduction might be attributed to changes in GDM-specific metabolic pathways, which could be linked to impaired glucose metabolism and insulin resistance in these individuals (Spanou et al., 2022).

In addition, our analysis enriched the tryptophan metabolism as the most significant pathway involved in GDM, and we found out that the level of the amino acid 5-hydroxytryptophan was dysregulated. 5-hydroxytryptophan is the upstream precursor of serotonin can increase serotonin levels (Paulmann et al., 2009). Serotonin may regulate insulin secretion from pancreatic β-cells by a mechanism of protein serotonylation (Paulmann et al., 2009). Since the 5-hydroxy-DL-tryptophan is downregulated in our GDM cohort, serotonin synthesis may not be promoted, thus not providing full protection from high glucose levels. Monitoring amino acid levels over time may represent a useful follow-up approach, given the evidence that such molecules might efficiently predict the transition from GDM to type 2 diabetes (Allalou et al., 2016).

Besides amino acids, lipid dysregulations have long been associated with glucose tolerance and insulin resistance (Ahmed et al., 2021; Giacco et al., 2019; Polsky and Ellis, 2015). Indeed, we found that many lipid molecules, including glycerolipids, glycerophospholipids, glycerophosphocholine, sphingolipids, steroids, fatty acyls, and their derivatives, were differentially represented in the GDM group as compared to the control one. Wang et al. reviewed that the major lipid classes subjected to quantitative alteration in GDM individuals are related to fatty acids, phospholipids, glycerolipids, glycerophospholipids, and sphingolipids (Wang et al., 2021). The quantitative dysregulation of fatty acids is almost considered a biochemical signature of GDM development, as such analytes are commonly associated with different components of insulin resistance and glucose metabolism in several studies (Bukowiecka-Matusiak et al., 2018; Ebbesson et al., 2010; Min et al., 2004).

Following our findings that highlighted the differential regulation of many lipid classes, other studies on GDM revealed that glycerolipids, glycerophospholipids, sterols, and sphingomyelins measured in plasma around the 10–14 weeks of gestation were correlated with increased GDM risk (Rahman et al., 2021). Even in the preconception phase, a metabolic signature identified a set of phosphatidylethanolamines (PE), a glycerophospholipids subclass built in the endoplasmic reticulum through the cytidine diphosphate-diacylglycerol-ethanolamine pathway, able to differentiate GDM from controls (Li et al., 2023). Mounting evidence has shown the active function of PE in the insulin signaling pathway, suggesting that the increased phosphatidylcholine (PC)/PE ratio can be correlated to reduced (Funai et al., 2016) or elevated (Newsom et al., 2016) insulin sensitivity among patients with type 2 diabetes. Recently, it was demonstrated that targeting phospholipid pathways improves insulin resistance in diabetic mice (Tian et al., 2023).

In such context, Anderson et al. discovered that multiple defects in lipid regulation (precisely including the significant changes in 4 phospholipids, 3 acylcarnitines, 3 fatty acids, and 4 diglycerides) may be identified in a pre-hyperglycemic phase before the occurrence of diabetes (Anderson et al., 2014). Another lipidomics study identified 10 dysregulated lipids in the serum of pregnant women that were significantly associated with impaired glucose tolerance; in particular, specific Triglycerides (TG), PC, and PCae were validated in an independent cohort as GDM predictor factors, independently on the maternal age or BMI (Lu et al., 2016).

More importantly, in our study, we identified a set of 10 metabolites, namely N-acetylproline, serylmethionine, sulfolithocholic acid, aminoadipic acid, hydroxyphenylacetylglycine, MG (0:0/16:1/0:0), deoxyadenosine monophosphate, dodecanoylcarnitine, indoxyl glucuronide, and 2-aminomuconic acid semialdehyde, whose combination showed high diagnostic value. With a high AUC of 0.978 (CI 0.898–1), this set of metabolites may represent a novel panel of biomarkers that can be tested to perform diagnosis of GDM with higher specificity than OGTT. We verified in our GDM cohort the diagnostic efficacy of the first two molecules (N-acetylproline, serylmethionine) with the highest frequency in the model we generated using ROC curves.

N-acetylproline (HMDB0094701) can be classified as a proteinogenic alpha amino acid L-proline derivative. Protein N-acetylation is a conserved post-translational modification that shields intracellular proteins from proteolysis, and N-acetyl amino acids can be obtained through either the direct synthesis by specific N-acetyltransferases or the proteolytic degradation of N-acetylated proteins by specific hydrolases (Perrier et al., 2005). It has been recently recognized that acetyltransferases might impact the development of early vascular and endothelial dysfunctions, prompting inflammation and oxidative stress (Di Pietrantonio et al., 2023). Currently, specific acetyltransferases are targets for therapy (Guo et al., 2021) or associated with the risk of diabetes (Al-Shaqha et al., 2015). Additionally, N-acetylation of many amino acids, including free proline, might produce uremic toxins when these molecules highly accumulate in serum or plasma (Tanaka et al., 2015; Toyohara et al., 2010). If not accurately filtered by kidneys, uremic toxins can lead to kidney insufficiency, cardiovascular affections, and neurological damage (Wang et al., 2021; Vanholder et al., 2008). Furthermore, N-acetylproline may be metabolically connected with the N-acetyl-seryl-aspartyl-lysyl-proline, an endogenous tetrapeptide with anti-fibrotic effects and clinical significance to combat kidney fibrosis in diabetes (Kanasaki, 2020). Given that N-acetylproline is significantly increased in GDM patients, our findings may be useful for the prediction of such complications in diabetes, especially during pregnancy (Wang et al., 2021).

Serylmethionine (HMDB0029045) is a dipeptide classified as a secondary metabolite. Based on the literature review, very few articles cite serylmethionine in their research. For example, it was employed as a substrate to investigate the function of peptidase enzymes, which show intense activity toward this metabolite during the first 2 weeks of life in rats (Vaeth and Henning, 1982). Up to the time of writing this manuscript, no studies have been published relating to the possible role of this molecule in the pathogenesis or the metabolism of GDM.

Despite the interesting findings of this study, recruiting a small number of patients from a single hospital would limit the generalizability of the findings and may appear to be a limitation. To assess the efficacy of the examined potential biomarkers in GDM diagnosis, varied cohorts with larger sample sizes and based on ethnic disparities should be considered. The metabolome is impacted by intrinsic (genetic mutations and epigenetics) and extrinsic (environment, diet, and stress) variables. As a result, intra-individual variances are magnified, significantly impacting the metabolomic profile. In future work, the potential of observed metabolic disturbances to predict adverse clinical outcomes of GDM should be studied. Furthermore, investigating metabolite-gene interactions will provide valuable insights into a better understanding of GDM.

## 5 Conclusion

This study provides valuable insights into the metabolic alterations associated with GDM by identifying significant differences in the metabolic profiles of pregnant women with and without GDM. The study identified 222 human endogenous metabolites significantly dysregulated in women with GDM. In addition, it highlights the dysregulation of key metabolic pathways, such as tryptophan, inositol phosphate, phenylalanine, and histidine metabolism, indicating specific pathways that could be targeted for early screening and intervention.

The study’s findings, particularly identifying key metabolites like N-Acetylproline and Serylmethionine, underscore the feasibility of using metabolic profiling as a diagnostic tool. This research contributes significantly to the growing knowledge of GDM, offering a promising avenue for early prediction and more effective management of the disorder.

## Data Availability

The original contributions presented in the study are included in the article/Supplementary Material, further inquiries can be directed to the corresponding authors.
